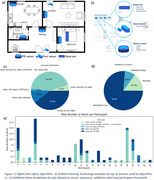# Monitoring night‐time safety in households with dementia using ambient sensing technologies: a rule‐based decision algorithm

**DOI:** 10.1002/alz70863_110614

**Published:** 2025-12-23

**Authors:** Alina‐Irina Serban, Sophie Horrocks, Chloe Walsh, Matthew Harrison, Eyal Soreq, Amer Marzuki, Mark Woodbridge, Ramin Nilforooshan, Rafael A Calvo, David J Sharp

**Affiliations:** ^1^ UK Dementia Research Institute, Care Research and Technology Centre, Imperial College London, London UK; ^2^ Department of Brain Sciences, Imperial College London, London UK; ^3^ Department of Mechanical Engineering, Imperial College London, London UK; ^4^ Helix Centre, Institute of Global Health Innovation, Imperial College London, London UK; ^5^ Surrey and Borders Partnership NHS Foundation Trust, Leatherhead UK; ^6^ Sorpol Consultancy, Ashdod Israel; ^7^ Dyson School of Design Engineering, Imperial College London, London UK

## Abstract

**Background:**

Dementia impairs cognitive abilities such as memory and decision‐making, significantly affecting activities of daily living. As the condition progresses, confusion, forgetfulness, and wandering become increasingly common, with wandering occurring in approximately 60% of people living with dementia (PLwD). Night‐time wandering poses substantial safety risks and increases caregiver burden. Despite its prevalence, there is a lack of effective tools to monitor and address these risks.

**Method:**

We developed a system that leverages ambient sensing technologies, including door sensors, in‐home motion detectors, and under‐mattress sleep sensors (Figure 1a‐b), combined with automated algorithms to monitor night‐time safety. Using a rule‐based decision model, the system analyses sequences of door usage and motion patterns to identify potential safety risks, such as leaving the house at night or leaving doors open. The algorithm underwent rigorous multi‐stage testing, including living lab evaluation, synthetic data simulation, retrospective analysis, and prospective validation.

**Results:**

Validation was conducted across 94 households over 365 nights, totalling 297,297 monitoring hours. At least one night‐time safety alert was triggered in 33 households, while no abnormal events were recorded in 61 households. Two hundred alerts were generated across 142 nights, with an average time outside 2 hours and 40 minutes per event. Of these alerts, 91.2% were validated as night‐time going out events, with 14.9% representing routine activities.

**Conclusion:**

This study demonstrates the algorithm's effectiveness in timely, accurate night‐time safety monitoring. Implementing it has the potential to enhance safety and reduce caregiver burden for PLwD in clinical and home care settings.